# A systematic approach to the disclosure of genomic findings in clinical practice and research: a proposed framework with colored matrix and decision-making pathways

**DOI:** 10.1186/s12910-021-00738-9

**Published:** 2021-12-25

**Authors:** Kenji Matsui, Keiichiro Yamamoto, Shimon Tashiro, Tomohide Ibuki

**Affiliations:** 1 Division of Bioethics and Healthcare Law, The Institute for Cancer Control, The National Cancer Center Japan, Tsukiji 5-1-1, Chuo-ku, Tokyo, 104-0045 Japan; 2 Office of Bioethics, The Center for Clinical Sciences, The National Center for Global Health and Medicine, Tokyo, Japan; 3 Department of Sociology, Graduate School of Arts and Letters, Tohoku University, Sendai, Japan; 4 Institute of Arts and Sciences, Tokyo University of Science, Noda-shi, Japan

**Keywords:** Genomic findings, Disclosure, Decision-making, Framework, Primary findings, Secondary findings, Incidental findings

## Abstract

**Background:**

Whether and how to disclose genomic findings obtained in the course of genomic clinical practice and medical research has been a controversial global bioethical issue over the past two decades. Although several recommendations and judgment tools for the disclosure of genomic findings have been proposed, none are sufficiently systematic or inclusive or even consistent with each other. In order to approach the disclosure/non-disclosure practice in an ethical manner, optimal and easy-to-use tools for supporting the judgment of physicians/researchers in genomic medicine are necessary.

**Methods:**

The bioethics literature on this topic was analyzed to parse and deconstruct the somewhat overlapping and therefore ill-defined key concepts of genomic findings, such as incidental, primary, secondary, and other findings. Based on the deconstruction and conceptual analyses of these findings, we then defined key parameters from which to identify the strength of duty to disclose (SDD) for a genomic finding. These analyses were then applied to develop a framework with the SDD matrix and systematic decision-making pathways for the disclosure of genomic findings.

**Results:**

The following six major parameters (axes), along with sub-axes, were identified: Axis 1 (settings and institutions where findings emerge); Axis 2 (presence or absence of intention and anticipatability in discovery); Axis 3 (maximal actionability at the time of discovery); Axis 4 (net medical importance); Axis 5 (expertise of treating physician/researcher); and Axis 6 (preferences of individual patients/research subjects for disclosure). For Axes 1 to 4, a colored SDD matrix for genomic findings was developed in which levels of obligation for disclosing a finding can be categorized. For Axes 5 and 6, systematic decision-making pathways were developed via the SDD matrix.

**Conclusion:**

We analyzed the SDD of genomic findings and developed subsequent systematic decision-making pathways of whether and how to disclose genomic findings to patients/research subjects and their relatives in an ethical manner. Our comprehensive framework may help physicians and researchers in genomic medicine make consistent ethical judgments regarding the disclosure of genomic findings.

## Introduction

In parallel with rapid developments and practical implementation in genomic technology, ethical questions with respect to genomic studies in medical research and clinical practice have been discussed on a global level over the past two decades. A relevant topic is whether and how to disclose genomic findings, particularly those of an incidental nature, to individual patients/research subjects. The first wave of debates came in the late 1990s in the immediate aftermath of the highly successful Human Genome Project [1]. However, the number of bioethics papers focused on this issue was quite limited until 2005, with the advent of next-generation gene sequencing devices of increasingly higher performance. The sudden ability to characterize readily the genetic variants of medical significance prompted a second wave of debate, especially during the mid-2010s. In 2013, the American College of Medical Genetics and Genomics (ACMG) listed 56 genes which they thought should be actively screened for and, if found, disclosed in clinical settings as “incidental findings” (IFs) [2]. However, as the ACMG’s recommendations were highly controversial and confusing, this listing elicited much criticism and debate [3], leading to the publication of several recently released governmental reports such as *“ANTICIPATE and COMMUNICATE”*, issued by the Presidential Commission for the Study of Bioethical Issues (PCSBI) [4, 5]. Despite extensive debate and policy-making efforts, no international consensus on the disclosure of individual genomic findings has been reached in research or clinical contexts [6].

Some organizations and expert groups, however, have developed recommendations and tools to address the issue. For instance, the Australian government created a *Decision tree for the management of findings in genomic research and health care* [5]; the UK Public Health Genomics Foundation provided a simple management chart [7]; the Boston Children’s Hospital Informed Cohort Oversight Board developed a disclosure flowchart that also includes participants’ preferences [8]; and the National Academies of Sciences, Engineering, and Medicine (NASEM) provided a conceptual framework with detailed recommendations [9]. Yet, none of these efforts have been sufficiently systematic or inclusive enough to enable all stakeholders in both research and clinical settings, including clinicians, medical genomic researchers, healthcare and research ethics committees, patients, and research subjects, to render consistently the ethical decisions regarding the disclosure of genomic findings, guided by well-defined ethical imperatives. Moreover, in most cases, the suggested protocols conclude with statements that researchers and/or their institutions and ethics committees should ultimately make their own decisions regarding whether and how to disclose individual genomic findings [9]. The conceptual underpinnings of key terms used in those documents, such as IFs, secondary findings (SFs), and unsolicited findings, overlap, but not entirely, and are therefore ill-defined, undermining appropriate decision-making. As a consequence, stakeholders are tossed about by a flood of fragmented, inconsistent, and equivocal guidance on disclosure, and left to bear ethical and sometimes legal responsibility for their decisions [10, 11]. Therefore, without optimal, easy-to-use tools for physicians and researchers working in genomic medicine that provide explicit directions for disclosure judgments, the growing conflict between emerging ethical imperatives may become unmanageable [12].

In this paper, we provide a chart illustrating the bases by which factors that contribute to the ethical balance for or against disclosure can be weighed and assessed in order to arrive at what we call the “strength of duty to disclose” (SDD) for each genomic finding. The process also addresses variants of unknown significance (VUS) obtained in the course of clinical practice or research. Here, our attempt is not to give a normative argument to justify decisions for disclosure/non-disclosure of a genomic finding [13], but, based on relevant previous arguments in the concerned literature, to align systematically each level of the SDD of genomic findings and propose a better practical model for handling them consistently.

The proposed SDD chart is organized along four axes: 1 through 4, which are defined in detail later. Using the chart, clinicians and researchers can arrive at a rational, primary judgment on whether to disclose incidental, as well as primary genomic findings (PFs) and SFs. We also present an effective package of pathways for the ethical treatment of such findings based on two other axes (5 and 6) to be defined later. The pathways allow for differing levels of expertise among physicians and researchers, the preferences of individual patients/research subjects, and consultation with ethics committees and/or ethics consultants. Thus, via a systematic approach, not only clinicians and researchers, but all stakeholders, including ethics committees and patients/research subjects, can be involved in order to arrive at consistently ethical disclosure decisions.

The present proposed framework, which consists of a multi-colored SDD chart and subsequent decision-making pathways, is developed partly based on our two preliminary studies published elsewhere, a conceptual analysis along with a targeted literature review of IFs [14], and an empirical exploration involving a focus group analysis of the concept of “actionability” [15]. The latter is a key concept critical for rendering disclosure decisions and judging the SDD of findings, as well as for providing an ethical basis for the treatment of genomic findings.

## Materials and methods

The original studies, including the published two preliminary studies, were conducted by members of the Ethics Task Force in the Nakagama Study Group (2014–2017) funded by the Ministry of Health, Labour and Welfare, and the Japan Agency for Medical Research and Development. The Ethics Task Force worked on the development of a clinical practice system, training, and education for genomic medicine at medical institutions. The Task Force consisted of the present four authors (who have expertise in clinical research ethics and bioethics, along with backgrounds in clinical and preventive medicine, philosophy, medical sociology, and ethics). In collaboration with physicians/researchers working on genomic medicine in the Nakagama Study Group, the Task Force was asked to create better methods for resolving ethical issues arising from the clinical application of genomic medicine, including how to address IFs and other genomic findings discovered in clinical and research settings. After the Nakagama Study concluded, the Task Force continued their investigations as the Matsui Ethics Study Group (2015–2019) under the auspices of the Japan Society for the Promotion of Science.

**Fig. 1:**
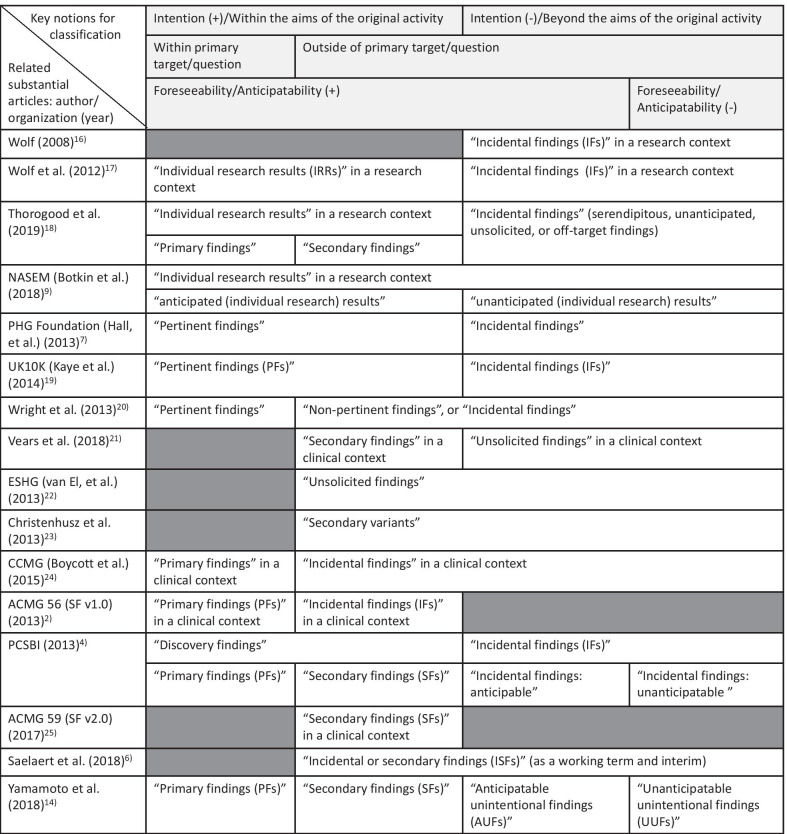
Terminology of genomic findings and previously proposed classification of findings

The Task Force had numerous face-to-face meetings over a 5-year period. During these meetings, the Task Force conducted a targeted literature review in order to parse and deconstruct the notions of PFs, SFs, IFs, and other types of findings (Fig. 1) [2, 4, 6, 7, 14, 16–25]. The Task Force also defined the six key parameters below, referred to here as “axes”, from which to identify the SDD of genomic findings from both clinical work and research studies. The Task Force also examined a variety of other proposed frames for the disclosure of genomic findings.

## Results

A conceptual analysis of the existing literature identified the following six major axes, along with sub-axes that, taken as a whole, provide a framework within which the SDD of each genomic finding (Axes 1–4) can be categorized, and the subsequent pathway for or against disclosure (Axes 5 and 6) may be systematically determined:

- Axis 1. Settings and institutions where findings emerge: (Axis 1–1) *clinical versus research settings*; (Axis 1–2) *advanced care hospitals versus primary care hospitals/small clinics*; (Axis 1–3) *research institutions with clinical departments versus research-only non-clinical institutions*; (Axis 1–4) *ordering physicians versus delegated genomic-testing laboratories*; (Axis 1–5) *primary researchers versus secondary researchers/users*
- Axis 2. Presence or absence of intention and anticipatability in discovery: (Axis 2–1) *intentional versus unintentional discovery*; (Axis 2–2) *anticipatable versus unanticipatable discovery*
- Axis 3. Maximal actionability at the time of discovery: (Axis 3–1) *treatment/prevention, diagnosis, or candidate screening for clinical trials*; (Axis 3–2) *non-medical and unknown action options*
- Axis 4. Net medical importance: *high, moderate, low, or unknown importance, or known in other areas of disease*
- Axis 5. Expertise of treating physician/researcher: *falling within versus outside of one’s domain of expertise*
- Axis 6. Preferences of individual patients/research subjects for disclosure

**Fig. 2:**
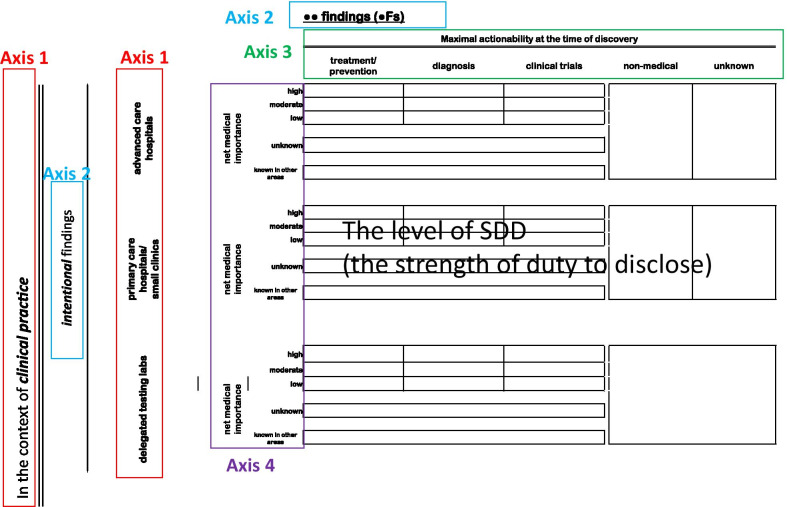
Relationship between Axes 1, 2, 3, and 4 (determinants of the SDD level)

The SDD of a genomic finding can be determined by the integration of four axes—Axis 1, Axis 2, Axis 3, and Axis 4 (Fig. 2). By our current definition, when a genomic finding is categorized in the colored matrix of the SDD as “had better be disclosed”, it means that the finding is considered as a finding with the highest disclosure level and thus, without exception, is to be disclosed. Likewise, when the finding is categorized as “must be disclosed”, it means that the finding has the second highest ethical demand for disclosure, unless there is some incontestable reason not to do so. Notably, there may be some debate about whether “had better” is a stronger expression linguistically than “must”, but for the present study, we would consider that “had better” is stronger, given its implication of the presence of a negative consequence or threat if the action is not performed, while “must” has no such implication [26]. On the other hand, when the finding is categorized as “should be disclosed”, it means that the finding should be generally disclosed, unless there exist strong reasons not to do so. The category “may be disclosed” means that disclosure is suggested, yet optional and at the discretion of the physicians or researchers. Lastly, “need not be disclosed” means that disclosure is entirely optional without any ethical appraisal. Axes 5 and 6 do not directly influence the categorization of the SDD of a genomic finding, as they relate to how to return the finding.

### Axis 1: Settings where findings emerge

#### Axis 1–1: Clinical settings versus research settings

It is now well-understood that ethics, and therefore the SDD, of a shared action or fact that emerges in clinical versus research settings may differ [27, 28]. That is, when within the original aim of genomic tests, a clinical physician who ordered the tests for their patient bears far more responsibility to disclose any PFs than a researcher does for their research subject [29], because the physician’s responsibility stems from their fiduciary relationship with the patient. Conversely, however, because of this relationship, findings obtained in clinical settings that lack therapeutic utility or significance, such as a variant of unknown significance (VUS), should not be actively sought per se [28, 30].

In contrast, within a research setting, even if a researcher may bear some responsibility to the subject to disclose certain types of individual findings, the SDD is generally much less than that in clinical settings because the source of responsibility to do so is simply found, as Miller et al. depicted, in obligations of general beneficence within the normative structure of a professional relationship [10]. This difference in SDD between clinical and research settings would remain even if it were based on other theoretical models, such as the classical duty-to-rescue model [28], or Richardson and Belsky’s partial entrustment model [31, 32]. The difference can also be described from a legal basis: while a physician in clinical practice is generally legally liable to disclose findings obtained in a purposed test (otherwise s/he may be charged for negligence), a researcher is generally not, except in extraordinary cases where a law mandates it [29, 33]. Therefore, it is reasonable to conclude that the SDD in research settings is generally lower than that in clinical settings [34].

#### Axis 1–2: Advanced care hospitals versus primary care hospitals/small clinics

The SDD may also differ by medical setting, such as one’s institution. Clinical physicians have different roles and responsibilities within institutional structures, irrespective of their intentions or personal values [35]. Generally, physicians at advanced care facilities, such as university hospitals and national medical centers, have greater access to resources, and may often be involved in more advanced medical practice, including clinical genomics [36]. Thus, they may be expected to have a greater degree of specialized expertise in their fields and bear greater responsibility for genomic findings than those at primary care hospitals or small clinics.

This is especially true, for instance, when genomic findings are used as eligibility criteria for trials involving an experimental drug against, for example, a rare cancer. While university hospitals or national medical centers generally have the infrastructure and staff expertise to conduct such trials, primary care hospitals or small clinics are less likely to. Given their more limited capabilities, the latter institutions should not seek such genomic findings, regardless of whether they are intended to serve in a primary or secondary manner. Accordingly, whether the institution is an advanced or primary care facility will alter the baseline SDD of a genomic finding. Nonetheless, if a primary care facility still opts to seek a critical genomic finding, they then bear an equal level of responsibility to an advanced care facility as to how they treat that finding.

#### Axis 1–3: Research institutions with clinical departments versus research-only non-clinical institutions

Generally, university hospitals or advanced medical research hospitals are institutions where clinical genomics is practiced daily and collaborations and communication between researchers and clinicians are expected. In such advanced institutions, genomic researchers may provide clinical services within their respective clinical genomics departments. If so, then genomic researchers in such institutions bear more responsibility toward a genomic research finding than those at non-clinical, research-only institutions because they may be involved in quasi-physician–patient fiduciary relationships. On the other hand, researchers at non-clinical, research-only institutions cannot be expected to bear the same level of responsibility [3], because research physicians in such institutions cannot legally provide clinical services. This includes the disclosure of genomic findings to research subjects for any clinical purpose or indication. If they still decide to do so, they must transmit such findings to a clinical department that exists apart from their institution, since whether even this step is legally permissible may be questionable. Needless to say, it is illegal to practice anywhere unless a researcher has a medical license. For these reasons, researchers in research institutions with clinical departments can be said to have a higher obligation to disclose findings than researchers at research-only, non-clinical institutions [33].

#### Axis 1–4: Ordering physicians versus delegated genomic-testing laboratories

In clinical settings, a patient’s physician decides which test (including genomic tests) to order, and the designated laboratory analyzes the given samples based on the physician’s order. Therefore, the designated laboratory has no direct responsibility to return any PFs or SFs to the patient: it simply has a duty to report the results of the analyses to the ordering physician, based on the assigned contract. However, beyond any individual physician’s orders, if the laboratory itself actively seeks other genomic findings, for example, SFs based on the latest ACMG’s list of genomic variants [25], then the laboratory’s autonomous activity in theory generates a responsibility to disclose such findings to the patient (even though the PF must go directly and only to the ordering physician). Thus, this bears the same SDD for additional laboratory findings as the patient’s physician has for PFs.

On the other hand, regardless of anticipatability (described later), an IF (AUF or UUF) may result from, for instance, a lab test ordered when the analyzer used is brand-new or recently upgraded, such that the lab staff can hardly anticipate all additional findings. In such circumstances, because the finding was discovered unintentionally, the SDD would be lower than that for PFs or SFs.

#### Axis 1–5: Primary researchers versus secondary researchers/users

In research settings, it often matters who discovers the genomic finding, whether by ‘primary researchers’ (defined here as any individual or group of collaborators) who shoulder the responsibility of asking a research subject for their permission to collect and use original samples from the subject, or by ‘secondary researchers or users’ who are provided with de-identified or anonymized samples from primary researchers [17].

Primary researchers always have a commitment to their research subjects because, as defined above, they are those who theoretically have had direct contact with the research subjects and are responsible under contract with them on the basis of informed consent. On the other hand, secondary researchers/users do not: they are simply provided data from primary researchers to use in the form of de-identified samples. Therefore, the SDD of a genomic finding discovered by secondary researchers/users is much lower than for that discovered by primary researchers. Accordingly, the SDD of findings obtained by secondary researchers/users is most often very low. If anything, they bear only a responsibility to return the finding to primary researchers to whom they have a committment, rather than disclosing it directly to the research subject.

### Axis 2: Presence or absence of intention and anticipatability in discovery

Based on bioethics literature such as the PCSBI report, it appears reasonable to distinguish levels of SDD according to whether findings are intentional or unintentional (Axis 2–1) and whether they are anticipatable or unanticipatable (Axis 2–2). In differentiating between these two axes and the levels of SDD, it may be helpful to reference the Principle/Doctrine of Double Effect. Based on this principle, it is ethically unacceptable for a person to intentionally cause bad consequences, whereas it is ethically acceptable for him to cause unintentional but anticipatably bad consequences [37]. It is true that moral philosophers have challenged the very idea of distinguishing the intended from the anticipated on that principle, arguing that it is impossible to tell them apart in a clear way. But it is also true that the principle has had a certain place in moral philosophy, which implies its significance in moral theories and reasoning [38]. Thus, we focus on these two axes—whether a finding is intended or not, and whether it is anticipatable or not—to understand how the SDD of a genomic finding can be determined by each axis.

#### Axis 2–1: Intentional versus unintentional discovery

The common feature between PFs and SFs is the notion of “actively sought” in the PCSBI report [4]. Since this notion can imply intention, the report assumes that it makes a morally relevant difference whether a finding is intentional or unintentional. Moreover, the report distinguishes PFs and SFs from unintentional findings or IFs in the broad sense [4]. As a result, IFs are regarded in the report as findings that are not actively or intentionally sought. On the other hand, in the original 2013 ACMG recommendations, which triggered heated arguments on IFs, IFs are defined as “the results of a deliberate search for pathogenic or likely pathogenic alterations in genes that are not apparently relevant to a diagnostic indication for which the sequencing test was ordered” [2]. ACMG then updated the recommendations in 2016, in which IFs and SFs are clearly distinguished, with the result that “the shift in terminology also maintained consistency with a recommendation by the Presidential Commission on Bioethical Issues” [25].

Not only from a legal perspective [39], but also from a moral point of view, one of the most important distinctions that make a relevant difference for moral judgment is whether an action is executed intentionally or not. Indeed, several moral theories hold good will or good intentions as the locus of moral evaluation [40]. Furthermore, according to one moral theory on intentionality, an intention is not merely a predominant desire for an action, but has the element of commitment to an action [41]. Given the above account, if a genomic finding is intentional, the action required to obtain the finding must involve some kind of commitment. Furthermore, from a moral point of view, it seems highly plausible that such an intention with commitment accompanies a certain moral responsibility, leading to moral duty or obligation. Thus, when one actively or intentionally seeks a genomic finding, that action carries a certain moral responsibility for the finding, and the seeker has a moral duty to disclose the finding. We consider this duty to disclosure to be a *“prima facie* duty”. A *prima facie* duty or a “conditional duty” refers to “the characteristic (quite distinct from that of being a duty proper) which an act has, in virtue of being of a certain kind (e.g. the keeping of a promise), of being an act which would be a duty proper if it were not at the same time of another kind which is morally significant” [42]. In other words, if one intentionally looks for a genomic finding, the seeker has a *prima facie* duty with a relatively weighty SDD to disclose that finding.

As implied by the PCSBI report and the ACMG’s updated recommendations, the most important moral determinant here is whether a finding is intentional or not. PFs and SFs are both intentionally sought, and thus have a stronger SDD than unsought findings. Yet, PFs and SFs do not have identical SDDs; the former may generally have a stronger SDD because they result from a stronger commitment or intention to seek them. Indeed, it is reasonable to assume that the intention to seek a PF is stronger than that for a SF, especially in the context of clinical practice. Therefore, adjunct to whether a finding is intended or not, the estimation of how strong the intention is would be another important element in rating the SDD. Thus, as shown in Fig. 1, the presence or absence of intention is a major element in rating the SDD of genomic findings.

#### Axis 2–2: Anticipatable versus unanticipatable discovery

IFs are classified into the anticipatable and unanticipatable in the PCSBI report. The report states that a finding is anticipatable when it “is known to be associated with a test or procedure” [4], regardless of its commonness or likelihood to occur. However, anticipatability may vary with the finder’s expertise and qualified specialized knowledge. That is, if an IF was anticipatable given a finder’s expertise and knowledge, then it is reasonable to assume that the finder could have anticipated the finding. Therefore, the finder has a stronger duty to disclose the finding. In contrast, an IF is unanticipatable if it falls outside of the finder’s expertise or if its discovery is not obvious in view of the current state of scientific knowledge in the scientific community to which s/he belongs [4]. In such cases, the finder has no duty to disclose the finding.

The difference in strength of the duty to disclose an IF on the basis of anticipatability differs categorically from that for intended findings, such as PFs and SFs—thus, the difference between Axes 2–1 and 2–2. Also, as per the PCSBI report, we can expect that, if IFs are anticipatable (AUFs), their finder may have a weak *prima facie* duty to disclose, whereas if they are unanticipatable (UUFs), then the finder may have no such duty.

In summary, with respect to Axes 2–1 and 2–2, we regard both PFs and SFs that are intentionally sought to categorically invoke a higher SDD than IFs (AUFs or UUFs). This conclusion is in line with what Eckstein et al. argues: “…we reject the distinction between primary and secondary findings…The central distinction between primary and secondary research findings is their nexus with research aims and objectives. …nexus with specific aims is not useful distinction” [43]. Yet, the SDD of PFs and SFs still differ, as discussed above. Thus, Axis 2–1 can be used to judge the level of SDD in practice. Perhaps one might even claim that only Axis 2–1 is needed to create an SDD flowchart. That is, unless a finding is determined to be an IF, the issue of anticipatability lacks relevance in assessing the SDD. We will review such distinctions in Axis 5.

### Axis 3: Maximal actionabilities at the time of discovering a genomic finding

While actionability is generally an important concept to manage findings, the types of actions may, in practice, vary widely. Some investigators argue that actionable options can also differ by institution [36], or by individual patients/research subjects or physicians/researchers [44]. However, such differences can be addressed largely via Axis 1, within the macro-distinctions of SDD and similarly via the practical pathway in treating individual findings described in Axes 5 and 6. Therefore, we focus here solely on plenary medical, non-medical, and unknown actionabilities as ways to treat findings. That is, the best available option or “maximal” actionability at the time of the discovery.

#### Axis 3–1: Treatment/prevention, diagnosis, or candidate screening for clinical trials, as medical actionability

The term “medical actionability” focuses on actionabilities judged by a medical professional [45]. Many scholars suggest that medical actionability yields only two options: disclosure or no disclosure [43, 46]. However, broadly speaking, medical actionability can be parsed into treatment, prevention, or settling a diagnosis [44]. Thus, the SDD of a genomic finding can vary depending on which of the above options is applied. In addition, physicians may feel that enrolling a patient in an experimental clinical drug trial is a medically actionable option, especially if there is no established therapy for the conditions indicated by the finding [15]. For example, in a clinical setting, if there is an established treatment/prophylaxis for Problem X indicated by Finding x, such an option is regarded as the maximal actionability at the time x was discovered. In this case, the ethical imperative to disclose x is stronger than if there is, as yet, no such established treatment/prophylaxis. Similarly, if there is a long-standing undiagnosed Problem Y that has not been treated or prevented effectively, but a newly discovered Finding y finally settles the diagnosis or corrects the initial diagnosis, then settling or correcting the diagnosis is seen as the maximal actionability pertaining to Finding y at the time of discovery [6]; and the ethical imperative to disclose Finding y is stronger than when Finding y could not confirm the diagnosis, but is only used to screen a potential research subject for a clinical trial [6]. Yet, the latter imperative may still be weaker than when an established treatment/prophylaxis for Problem Y exists, unless other overriding factors are present.

Based on these considerations as well as our previous findings [15], we can classify the best available, or “maximal”, medical actionability options toward a genomic finding at the time of its discovery into three categories: (a) established treatment or prophylaxis (“treatment/prevention” option); (b) there is as yet no useful treatment or prophylaxis, but a useful diagnosis, prognosis, or prediction of the course of the disease is possible (“diagnosis” option) [47]; and (c) established treatment/prophylaxis or useful diagnosis options are not available yet, but the finding can be used for a candidate screening index for clinical trials (“clinical trials” option). The SDD can then vary, depending on which of these three maximally actionable options is available. When the treatment/prophylaxis option is the best available option at the time of discovery, then its SDD is rated highest. The SDD will be lower when the diagnosis option is the maximally actionable option at the time of discovery, and lowest when the best actionable option is the clinical trials option.

#### Axis 3–2: Non-medical and unknown action options

Even if none of the above-mentioned medical actionability options apply to a finding, patients or research subjects may still accrue considerable personal benefit by having an important finding disclosed to them. Such non-medical actionability involves, for instance, the potential for psychological preparedness via “knowing”, or reproductive decisions and other important life plans [44, 48]. However, the non-medical options that are best for a person at the time of discovery may inevitably vary according to the views and attitudes of each individual [49], and therefore it is impossible for physicians/researchers to choose disclosure/non-disclosure in advance based on the given SDD of such non-medical options. Thus, such individual views of value should be considered *later* in the decision-making process as reflected in Axis 6.

Findings that are variants of clinically or scientifically unknown significance (VUS) have, by definition, no currently medically actionable option, and therefore no personally actionable option either. Accordingly, the SDD of a VUS is generally very low and not worthy of being disclosed in most cases. In part, this is because clinical physicians generally would not *intentionally* seek VUS, as they lack clinical meaning, except in very rare instances, e.g., when a VUS finding may anchor a candidate’s screening for a clinical trial. However, if there is any clinically clear reason or necessity for physicians to believe that they must look for a certain VUS as a primary objective or a secondary objective, then the act of seeking a VUS would inevitably increase the SDD. On the other hand, in research settings, researchers often intend to seek a VUS for primary or secondary research purposes because they wish to examine the meaning of a possible function and an expected role by the VUS. They examine the VUS because its scientific and clinical importance is still unknown. In such cases, researchers have no obligation to disclose a discovered VUS, unless they have promised to do so.

### Axis 4: Net medical importance judged by responsible professionals

In order to assess the best follow-up action for responding to a genomic test result indicating the presence of a known pathogenic variant, a physician must weigh whatever medical option seems best for the patient’s individual health against broader medical or clinical concerns surrounding the patient’s condition. Weighing such choices involves at least an assessment of test accuracy (i.e., analytic validity), clinical validity of a detected/predicted illness [43, 48, 50], and clinical urgency (or anticipated age of onset, especially for children) [15, 51–53]. When the perceived condition lies within one’s own domain of expertise, a physician can render a professional judgment as to net medical importance. Even if the problem is beyond one’s immediate expertise, a physician can often estimate the net medical importance by reviewing relevant medical literature. Subsequent to these considerations [4], a physician may render a final judgment of net medical importance, that immediate or deferred treatment is *highly*, *moderately*, or *less* clinically important, or that other specialists should be consulted. However, if the test result indicates only a VUS with no established treatment or medical protocol, then, of course, the medical importance of the VUS remains unknown, and the physician generally has no further clinical role.

Similar considerations and subsequent professional judgment of net medical importance of indicated conditions can also be expected in research settings. This is especially true if the genomic researchers are also physicians, or when the indicated finding is within the domain of their expertise, even if they are not physicians.

### Development of a colored SDD matrix of discovered genomic findings

**Fig. 3:**
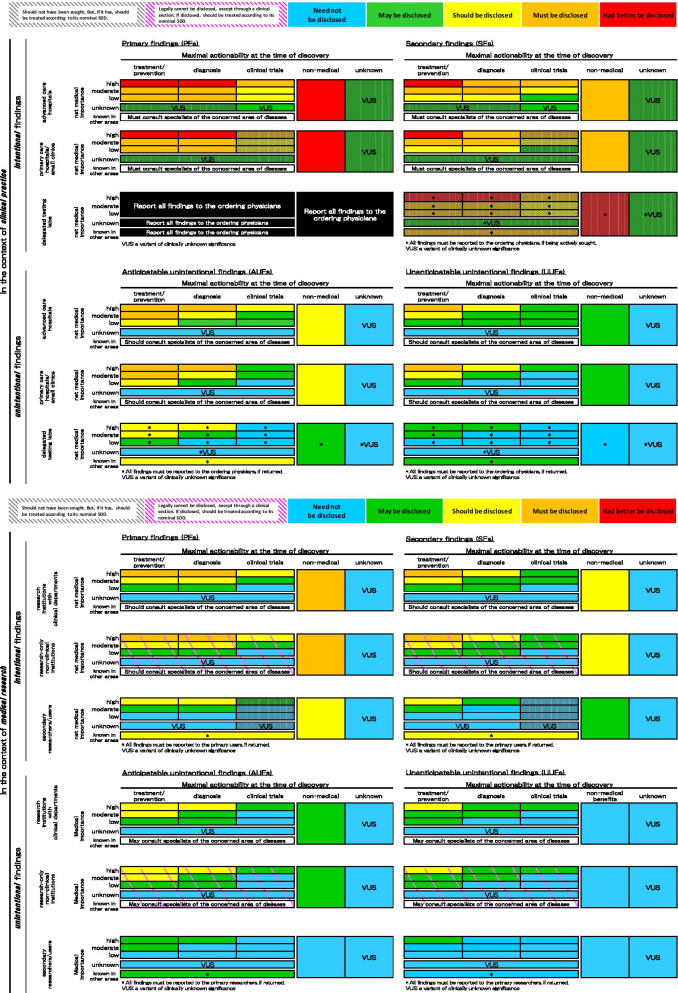
A model multi-colored matrix of the strength of duty to disclose (SDD) of genomic findings

Using the four axes discussed above, we developed the multi-colored matrix in Fig. 3, which depicts one possible scheme for assessing the SDD of individual genomic test results, i.e., PFs, SFs, AUFs, and UUFs [14], in clinical and research settings. The following decision matrix reflects the consideration of each context and circumstance that results in a genomic finding, and is composed of five levels of SDD, including *‘had better be disclosed* (red)*’*, *‘must be disclosed* (orange)*’*, *‘should be disclosed* (yellow)*’*, *‘may be disclosed* (green)*’*, and *‘need not be disclosed* (light blue)*’*. Any colored cell covered with black oblique lines means that a finding in that cell *‘should not have been sought’*, but if it has, then the finding should be treated according to its nominal SDD. Likewise, any cell covered with pink oblique lines indicates that a finding in that cell, regardless of nominal SDD, *‘cannot legally be disclosed, except through a clinical department’*, and if disclosed, should be treated according to its nominal SDD.

In determining the SDD category of each cell, we applied first and foremost “had better to be disclosed” to the category of those cells of PFs obtained in the clinical practice which have high net medical importance and have either the treatment/prevention option or the diagnosis option, because we considered no one would disagree with this categorization for those specific cells. Using them as the absolute standard, each relative SDD category of other cells was determined reasonably by the considerations of Axes 1 through 4 as well as the sub-axes (see above description). When direct determination of the category of a cell was difficult based on those axes/sub-axes, it was determined through a conference of four authors.

### How to use the colored SDD matrix

BRCA1 is currently one of the most well-known pathogenic genomic variants of clinical significance. It is listed in the ACMG SF v3.0 and analogues as a finding highly recommended to be disclosed because it indicates breast/ovarian cancer risk of high medical importance [54, 55], and because there is an established treatment/prevention protocol for breast/ovarian cancers. If BRCA1 is sought as a PF in clinical settings, regardless of what hospitals or clinics planned to test, its SDD level is “had better be disclosed (to the patient)”. On the other hand, if the very same BRCA1 is discovered unintentionally as an AUF in a research setting, its SDD will be much lower, i.e., the “should be disclosed” category. This means that the finding should be disclosed if there is no compelling reason not to. If BRCA1 is discovered by a secondary user as part of their research, a BRCA1 finding is placed merely in the “may be disclosed” category. If, instead, it is discovered at a research-only institution without any clinical department, a BRCA1 finding may not legally be disclosed other than via a clinical department of a hospital or clinic.

Now consider a genomic finding X of known, moderate medical importance, for which the known maximal actionability at the time of discovery is limited to candidate screening for a clinical trial. If X is obtained as an SF in an advanced care hospital with sufficient competency in clinical trials, its SDD is marked as “should be disclosed”. However, the same finding X should not be sought by a small clinic that lacks competence in conducting clinical trials. Nonetheless, if such a clinic does happen to seek X as a PF or SF, the clinic must then bear the same level of responsibility for disclosure and subsequent follow-up as an advanced care facility. On the other hand, if X is sought as a PF or SF in a research setting, its SDD will be much lower than in a clinical setting; i.e., it would be rated as, “may be disclosed” or “need not be disclosed”.

Our SDD decision flowchart may also be applied to blood relatives of patients/subjects. To most such blood relatives, a genomic finding is categorized as an AUF, rather than a PF or an SF. Therefore, the SDD of BRCA1 for blood relatives, discovered via primary or secondary testing of a patient/subject, will be at most equal to, but almost always weaker, than the SDD of the finding for the patient/subject her/himself.

As our proposed SDD chart is merely a general map of the ethical landscape for the treatment of genomic findings, the specifics of individual cases must be completed via mapping with known pathogenic genomic variants in each disease area by experts or expert panels and be updated regularly. However, one benefit of utilizing our SDD chart is that it could standardize the handling of genomic findings discovered in clinical and research settings. Thus, it may enable physicians/researchers to render consistent ethical assessments of the SDD of genomic findings, such that they can proceed to make judgments on whether to disclose, as described in the next section.

### Decision-making processes with Axis 5 (within/outside of one’s expertise) and Axis 6 (preferences of individual patients/research subjects)

**Fig. 4:**
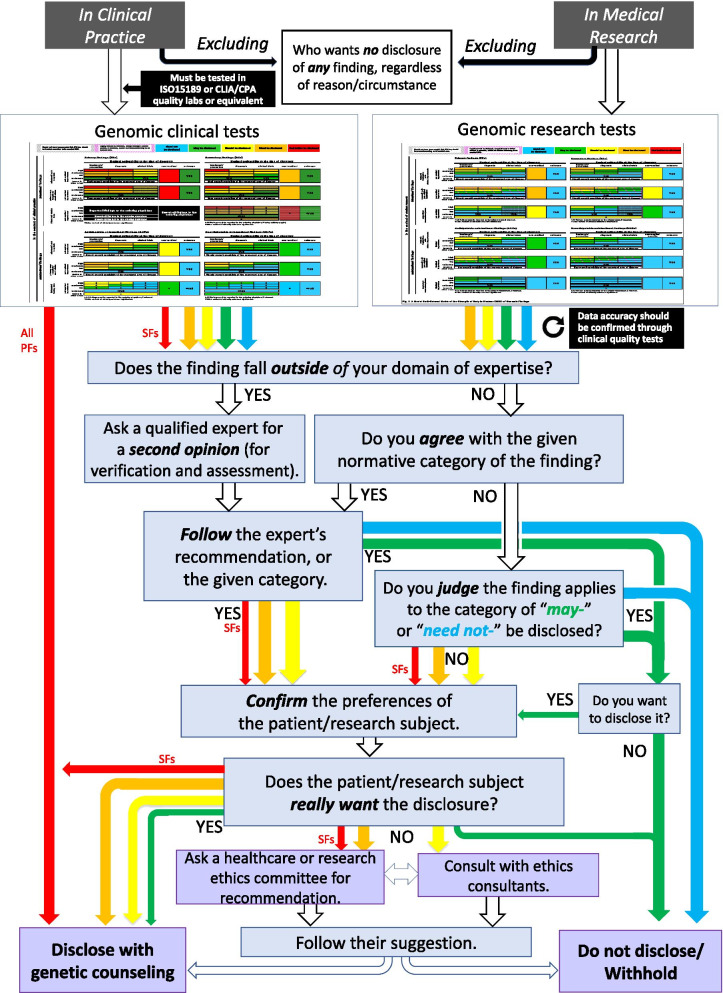
A model decision pathway for the disclosure/non-disclosure of a genomic finding

Once the SDD of an obtained finding is assessed via the colored SDD matrix in Fig. 2, physicians/researchers can then render a final decision regarding disclosure, after considering the patient/subject’s preferences (Fig. 4). This excludes findings from research venues, which should be affirmed in a clinically valid manner before rendering any final decisions.

Needless to say, guaranteeing a patient/subject’s “right-not-to-know” requires that they really, at the initial consent process and prior to the initial clinical testing or research involvement, are completely unaware of any possibility of non-PFs. A large body of evidence suggests that some people prefer not to know and are thus strongly adverse to disclosure [56–59]. In such cases, once physicians/researchers confirm their initial irrevocable non-disclosure preference for any additional finding, for instance, through a pretest genomic counseling, before enrolling them into genomic clinical testing or genomic research testing, or reconfirm their final non-disclosure preference for any non-PF after making the judgment of the finding’s SDD, no further decision processing is truly needed. However, in reconfirming a non-disclosure preference, several institutions now provide an ethics consultation service [60, 61]. Even if one’s institution does not have such a service, most institutions have an ethics committee that may provide some guidance [3, 62]. Therefore, physicians/researchers are recommended to seek actively such services when they have difficulty reconfirming or they have reasons to doubt a stated final non-disclosure preference.

With the exception of such strong non-disclosure instances, most patients/subjects would likely have a somewhat positive preference for disclosure [56–59]. If so, to what extent should physicians/researchers follow a patient’s initial desire indicated prior to testing? It is desirable at this point to assess whether it is actually appropriate to disclose the obtained finding based on their indicated initial desire, and also whether disclosure really aligns with the patient/subject’s true preference.

Yet, instances where a physician/researcher can render a responsible judgment for disclosure/non-disclosure may be quite limited, particularly when the obtained finding is beyond their area of professional expertise [3, 63]. In such cases, the responsible physician/researcher should seek a second opinion from a specialist. After going through these procedures, the findings judged as “may be disclosed”, “should be disclosed”, or “must be disclosed” will be disclosed, along with genomic counseling for those patients/subjects who desire it.

On the other hand, as shown in the colored SDD chart, the finding categorized as “had better be disclosed” can arise only in clinical settings. Without exception, PFs in this category must be disclosed along with genomic counseling, and without involving any of the above-mentioned intervening processes, because obtaining the genomic finding was the primary purpose of the physician’s treatment for the patient. Thus, disclosure of such PFs is an absolute requirement. However, SFs in this category should follow the above-mentioned processes, because obtaining SFs is not the primary purpose of the treatment, even though seeking them is within the discretion of the treating physician. Moreover, if the patient does not desire the disclosure of SFs, the responsible physician should seek the advice of the hospital ethics committee or a return-of-result-reviewing committee, if available [64]. Similarly, when a patient does not desire disclosure of findings in the “must be disclosed” category, it is good practice for the responsible physician/researcher to consult the ethics committee, regardless of how the discovery occurred or any categorical difference between PFs and SFs. Figure 4 provides a general picture of the whole process of deciding on whether to disclose a finding, as described above.

## Discussion

As our SDD chart indicates, the SDD of a genomic finding in clinical settings is generally higher than in research settings because clinicians have a legally binding, fiduciary duty to treat and care for their patients, while researchers do not. Therefore, for most cases in clinical practice, PFs and SFs that have some medical significance, along with some actionability options, should be disclosed, unless there are other compelling reasons not to do so. On the other hand, even if identical PFs or SFs are obtained in a research setting, their SDDs are generally much lower than in a clinical setting, except in cases where the finding has high medical significance, along with clear, medically actionable options. Moreover, researchers at research-only institutions without clinical departments must not run a research protocol that involves PFs/SFs obtained for screening purposes in clinical trials, since they are legally prohibited from performing any type of medical treatment.

Compared to intentionally sought PFs/SFs, AUFs or UUFs have quite limited SDDs, regardless of the clinical or research setting. Yet, without due consideration of such categorical differences in SDD, statements from professionally accredited sources often prescribe some blanket obligation to return such findings. However, as McGuire et al. and Pike et al. warn [33, 34], the proliferation of such statements will inevitably drag medical professionals into increased risk of legal jeopardy or even sanctions, even though, in most cases, they may not, in fact, be ethically obligated to return such findings. What has also been overlooked in the literature is that the moral obligation to disclose genomic SFs to kinfolk is much weaker than that to the patient/subject. Indeed, disclosure of SFs to blood relatives should be on par with that of AUFs, not SFs.

As for secondary researchers who simply use genomic samples and data provided by primary researchers, their ethical obligation for disclosure is much weaker than that of primary researchers. They are obligated to return findings only to primary researchers and/or their institutions, not to research subjects directly.

Our proposed SSD chart should be considered as something dynamic, rather than static, and should be updated regularly [17, 65], ideally by a review committee with expertise in each disease area as new genomic variants are discovered and their significance determined. One advantage of the proposed format is that even as variant classifications evolve and new actionability options become available, physicians/researchers can easily refer to an updated chart to judge the SDD for potential disclosure of variants of concern.

Through the regular use of our proposed framework consisting of the SDD chart with the flowchart of the subsequent decision-making pathways, decisions regarding how to ensure the ethical reliability of an assessment, how to respect individual preferences regarding disclosure, and how to ensure procedural justice, will lean toward reasonably systematic, rather than haphazard or inconsistent, judgments. We anticipate that our proposed framework would be helpful in providing physicians/researchers working in genomic medicine with a solid, systematic model approach to the complicated ethical issue of whether and how to return and disclose genomic findings.

One possible limitation of our framework would be that if a time-dependent view of ‘actionability’—namely, “[w]hat might not be actionable now may become actionable in the future” [21]—should be taken into consideration for the judgment of the level of SDD, our SDD matrix structured with available actionable options at the point in time of first discovery of a genomic finding might lose much of its relevance. Similarly, it may not be fully applicable to findings obtained in an epigenetic testing, where key notions in general genomic medicine such as ‘clinical validity’ or ‘actionability’ are not yet well established while epigenetic-specific notions of variants are getting conceptualized [66]. Also, our framework as it is currently may not be directly applicable to the practice of the direct-to-consumer (DTC) genomic testing [67], as DTC testing lacks any third-party initiator or orderer of the test other than the consumer him/herself. However, because DTC providers can be considered to bear the same responsibility to an ordering consumer as what the delegated testing labs in our chart owe to the ordering physician, the remaining parts of our framework may be applicable, even to DTC testing. Lastly, if the maximum ethical weight should be placed on respect for patient/subject autonomy, or if the patient/subject’s right for disclosure or the duty to disclose a genomic finding to patients/subjects should be legislated, our SDD matrix might diminish most of its usefulness.

## Conclusion

Based on the conceptual analyses of genomic results including PFs, SFs, and IFs (AUFs and UUFs), we studied the SDD of those findings, and the subsequent systematic decision-making pathway of whether and how to disclose them ethically to patients/subjects and their blood relatives. The present article is intended to develop a comprehensive model framework for judging the disclosure/non-disclosure of genomic findings discovered in the context of both clinical practice and research, and to provide a reasoned, yet optimal, easy-to-use management tool for decision-making for all stakeholders in genomic medicine.

Although our proposed framework is developed primarily with consideration of the circumstances surrounding genomic medicine and research in Japan, we hope that it may also be applicable or helpful to other countries where the issues of whether and how to return genomic findings responsibly to individuals, while respecting their preferences, rrepresent similar problems in medicine. Even if this is not the case, we still believe that it will serve as a model with a reasonable approach to the matter of our concern. One of the advantages of our framework is that it was developed based on ethical theories, previous rich arguments in literature, and practical applicability perspectives, enabling a reasonably systematic approach to the complicated ethical issues of disclosure of genomic findings. By maintaining and frequently updating the latest lists of known pathogenic findings to be entered into the SDD matrix, while being reviewed by specialists in each medical field, physicians/researchers working on a daily basis with patients and research subjects may make consistent, ethical judgments regarding the disclosure of PFs, SFs, and IFs, including AUFs and UUFs.

## Data Availability

Not applicable.

## Abbreviations

ACMG: The American College of Medical Genetics and Genomics
AUF: Anticipatable unintentional finding
CCMG: The Canadian College of Medical Geneticists
DTC: Direct-to-consumer
ESHG: European Society of Human Genetics
IF: Incidental finding
NASEM: The National Academies of Sciences, Engineering, and Medicine
PCSBI: The Presidential Commission for the Study of Bioethical Issues
PF: Primary finding
SDD: Strength of duty to disclose
SF: Secondary finding
UUF: Unanticipatable unintentional finding
VUS: Variant of unknown significance
